# Divergent immuno-landscapes: comparing the tumor microenvironment of Indian and Western HNSCC patients

**DOI:** 10.3389/fcell.2026.1725978

**Published:** 2026-04-23

**Authors:** Prashant Kumar Rai, Bibha Choudhary

**Affiliations:** 1 Manipal Academy of Higher Education, Manipal, India; 2 Institute of Bioinformatics and Applied Biotechnology, Bengaluru, India

**Keywords:** cancer, HNSCC, immunotherapy, Indian and Western population, tumor microenvironment

## Abstract

Head and neck squamous cell carcinoma (HNSCC) is a highly dynamic and heterogeneous cancer where the tumor microenvironment (TME) and immune landscape differ across populations. Western groups often show an “immune-hot” profile with infiltration by various immune cells, including cytotoxic CD8^+^ T cells, helper CD4^+^ T cells, and regulatory T cells. In contrast, Indian groups frequently display an inflammation-rich yet immunosuppressive microenvironment dominated by myeloid cells. The Asian population, particularly Indian patients, often exhibits distinct immune subtypes influenced by region-specific factors such as tobacco and areca nut use, rather than smoking and alcohol. Indian oral squamous cell carcinoma (OSCC) of the gingivobuccal region shows unique distributions and functional states of antitumor immune populations, with differences in activation and suppression signatures that impact prognosis, metastasis, and treatment response. Major Histocompatibility Complex (MHC) molecules are essential for the immunotherapeutic response, and studies have reported the influence of specific Human Leukocyte Antigen (HLA) class I and II gene variants on antigen presentation and T cell activation. These allelic variants may serve as indicators of tumor microenvironment (TME) involvement and could affect the effectiveness of immunotherapy. Recognizing population-specific variations in immune cell infiltration highlights biological differences in carcinogenesis and offers opportunities to discover new biomarkers and stratify patients for personalized treatment strategies.

## 1 Introduction

Head and neck cancer (HNSCC) is a complex disease affecting multiple areas of the head and neck, including the pharynx, larynx, tongue, oral cavity, tonsils, hard and soft palate, and paranasal sinuses. HNSCC ranks as the sixth most common cancer worldwide and poses a significant health challenge, impacting hundreds of thousands annually and greatly contributing to cancer-related morbidity and mortality (Desai et al., 2021). According to GLOBOCAN 2022, India plays a major role in the global burden of HNSCC. Among Asian countries, India accounts for 55.6% of lip and oral cavity cancer cases, 49.3% of oropharynx cancer, 48.5% of hypopharynx cancer, and 33.6% of larynx cancer (Sung et al., 2021). Lip and oral cavity cancers are the second most common in both incidence and mortality in India, and the burden of HNSCC is expected to rise significantly, with estimates indicating a 58% increase in new cases by 2040. Major risk factors include alcohol use, tobacco consumption, poor diet, inadequate oral hygiene, and viral infections such as human papillomavirus (HPV) and Epstein-Barr virus (EBV) (The Cancer Genome Atlas Network, 2015; Argiris and Eng, 2003). Recent studies also suggest that periodontal pockets may contain HPV, and chronic periodontitis could contribute to the development of tongue cancer (Hormia et al., 2005; Yu et al., 2002). Globally, Southeast Asia exhibits the highest incidence rates, followed by Europe and South America (Cheong et al., 2017). HNSCC features complex molecular and immunological interactions within its tumor microenvironment (TME). The TME plays a key role in tumor initiation, progression, and response to therapy (Das et al., 2024). Studies have shown that Western and Asian populations differ significantly in the causes and genomic features of HNSCC. In India, the high rate of gingivobuccal cancer is closely linked to chewing tobacco, areca nut use, and other factors, resulting in a distinctive immunogenic profile in HNSCC. Research using TCGA datasets has categorized Western populations into several subgroups with different levels of immune cell infiltration and checkpoint expression (Das et al., 2024). Immunotherapy has become an important treatment option, with the potential to boost anti-tumor immune responses. Recent research shows positive effects of immunotherapies on survival outcomes in both Indian and Western populations. This review compares the TME, immune landscapes, and HLA diversity between Indian and Western groups, highlighting differences in immune cell makeup and their potential effects on immunotherapy.

## 2 Tumor microenvironment in HNSCC

The HNSCC tumor microenvironment (TME) is a complex and continuously evolving system made up of tumor cells, immune cells, stromal cells, secreted proteins, and extracellular matrix components. These elements interact closely to create an immunosuppressive environment that promotes tumor growth and resistance to therapy (Das et al., 2024). The HNSCC TME contains many immune cells and non-cellular components. The cellular components include stromal cells such as cancer-associated fibroblasts (CAFs), blood endothelial cells, and lymphatic endothelial cells; immune cells such as CD8^+^ T cells, naïve and memory CD4^+^ T cells, T follicular helper cells (Th1, Th2), Tregs (nTreg, iTreg), gamma delta T cells, resting and activated NK cells, monocytes, macrophages (M0, M1, M2), resting and activated dendritic cells, mast cells, mucosal-associated invariant T (MAIT) cells, eosinophils, and neutrophils. The immunosuppressive conditions within the TME also involve MHC downregulation and impaired antigen-processing machinery, which hinder tumor antigen presentation, along with increased expression of checkpoint inhibitors on tumors and immune cells. These cellular components and molecular events highlight the immunosuppressive effects of the TME, which are essential to understanding the underlying mechanisms (Ferris et al., 2006; Lopez-Albaitero et al., 2006). These diverse cell populations work together to regulate immune activation, immune suppression, and tumor progression within the microenvironment.

## 3 Immune cell components of the TME

### 3.1 T lymphocytes

T cells constitute a significant part of the tumor-infiltrating lymphocytes (TILs) in HNSCC. Both HPV-positive and HPV-negative tumors show diverse infiltration of T cell subsets. CD8^+^ cytotoxic lymphocytes (CTLs) play a crucial role in antitumor immunity by recognizing and eliminating malignant cells through cytotoxic activity (Bhat et al., 2021). Multiple studies indicate that HPV-positive HNSCC exhibits greater infiltration by CD8^+^ T cells and other immune cells than HPV-negative HNSCC. This greater immune cell infiltration is linked to improved overall survival in HPV-positive HNSCC (The Cancer Genome Atlas Network, 2015; Ang et al., 2010; Fakhry et al., 2017). In HNSCC, TCR repertoire features have become important clinical biomarkers. Greater TCR diversity (richness) is associated with better therapeutic response and survival with cetuximab and nivolumab (Wang et al., 2025), while changes in clonality during treatment with immune checkpoint inhibitors such as pembrolizumab are linked to patient outcomes (Paschold et al., 2025). Additionally, antigen-driven expansion of high-affinity TCRs—supported by dendritic cell priming—indicates that dominant clonotypes represent tumor-specific immune responses rather than bystander infiltration (Zhang et al., 2025).

### 3.2 B lymphocytes

B cells are an essential component of the TME and contribute to tumor defense through various mechanisms, including producing tumor-specific antibodies, presenting antigens, and forming tertiary lymphoid structures (TLS). Specialized subsets, such as activated B cells (ABCs), memory B cells, and plasma cells, have been identified in the TME and perform distinct immune functions. In some cases, B cells make up about 40% of tumor-infiltrating lymphocytes (TILs) in Western HNSCC populations. These cells produce anti-HPV antibodies and other tumor-associated antigens (Conarty and Wieland, 2023; Jumaniyazova et al., 2022; Welters et al., 2018).

### 3.3 Natural killer cells and other lymphoid cells

Natural Killer (NK) cells are essential parts of the innate immune system and can eliminate tumor cells without prior antigen-specific priming. The Western HNSCC population shows a strong infiltration of CD56dim NK cells, which are known for their potent cytolytic activity. High levels of NK cell infiltration, particularly of CD56^dim^ NK cells, within the TME are associated with improved patient survival (Mandal et al., 2016). Studies suggest that HPV-positive tumors have a greater abundance of NK cell subsets. The presence of NK cells in the TME indicates both immune activation and potential immune exhaustion. Other lymphoid populations, such as gamma-delta T cells and invariant natural killer T (iNKT) cells, are also linked to antitumor immune responses (Galvani et al., 2026).

### 3.4 Myeloid cells and dendritic cells

Myeloid cells, including macrophages, monocytes, and myeloid-derived suppressor cells (MDSCs), play a vital role in the tumor microenvironment (TME). These cells act as key regulators that connect the innate and adaptive immune systems (Biswas and Mantovani, 2010; Dhodapkar et al., 2008; Guilliams et al., 2014). Tumor-associated macrophages (TAMs) can display different functional phenotypes. The proinflammatory M1 phenotype is associated with antitumor activity, whereas the immunosuppressive M2 phenotype promotes tumor growth (Mazilu et al., 2021). An increased presence of M2 macrophages in the TME is associated with a poor prognosis (Mandal et al., 2016; Marcus et al., 2004; Wolf et al., 2015). MDSCs support immune suppression by expressing high levels of CD68, PD-L1, and TGF-β, while also inhibiting IFN-γ expression and the proliferation of activated T cells (Chikamatsu et al., 2012). Dendritic cells (DCs) are central to antigen presentation and T cell priming. Both plasmacytoid DCs (pDCs) and conventional DCs are present, with mature DC subsets localizing near tumor nests in immune-inflamed tumors. Their capacity to prime T cells highlights their importance as potential targets for vaccine-based or adjuvant immunotherapies (Galvani et al., 2026). Higher dendritic cell infiltration has been observed in patients without metastasis compared to those with metastatic disease (Kikuchi et al., 2002). In HPV-associated oropharyngeal squamous cell carcinoma, cDC2 has been shown to play a key role in activating tumor-infiltrating T cells and promoting anti-tumor immune responses. In HPV-negative cancers, increased dendritic cell infiltration has been associated with improved clinical outcomes (Santegoets et al., 2020).

### 3.5 Cancer-associated fibroblasts (CAFs)

CAFs are a major non-hematopoietic component of the TME. These cells promote tumor growth, spread, therapy resistance, and support cancer stem cells. CAFs influence immune responses by releasing inflammatory cytokines, chemokines, and extracellular matrix components that regulate immune cell recruitment and activation (Bhowmick et al., 2004; Gascard and Tlsty, 2016; Kalluri, 2016; Kraman et al., 2010; Liotta et al., 2015; Orimo et al., 2005; Turley et al., 2015).

## 4 Indian and Western HNSCC immune landscapes

### 4.1 Indian immune landscape (predominantly HPV-ve HNSCC)

The immune landscape of HNSCC varies significantly between Indian and Western populations (Table 1).

**TABLE 1:** Comparative immune and clinical features of Western vs. Indian HNSCC.

Feature	Western population	Indian population
Etiology	HPV, smoking	Tobacco, areca
TME type	Immune-hot	Inflamed but suppressive
Dominant cells	CD8^+^, NK	M2 macrophages, MDSCs
Cytokines	IFN-γ dominant	IL-6, IL-8, TGF-β
Metabolism	Oxphos	Glycolytic
Immunotherapy response	Better	Limited

These differences are mainly due to variations in HPV prevalence, environmental exposures, and underlying genetic and epigenetic factors. In Indian populations, the immune landscape of HNSCC is marked by a low rate of HPV infection and predominant exposure to tobacco and areca nut-related carcinogens, resulting in a tumor microenvironment (TME) that is inflamed yet immunosuppressive. At the molecular level, this heterogeneity is reflected in distinct immune subtypes within Indian HNSCC groups. Transcriptomic studies by Das et al. (2024) identified two primary subtypes, GB1 and GB2. GB1 shows low immune infiltration, while GB2 is characterized by increased infiltration of CD4^+^ T cells, CD8^+^ T cells, B cells, and pro-inflammatory M1 macrophages. In the GB2 subtype, CD8^+^ T cells are more prevalent compared to the CD4^+^ helper and regulatory subpopulations (Das et al., 2024). Although HPV-positive tumors are relatively rare in Indian cohorts, they offer important insights into immune activation states. HPV-positive HNSCCs tend to have an inflamed, or “hot,” tumor microenvironment characterized by increased cytotoxic T cell infiltration. Despite this immune activation, the immune microenvironment’s functional state usually remains suppressed in most Indian HNSCC tumors, driven by increased M2 macrophages and myeloid-derived suppressor cells (MDSCs), elevated immunosuppressive cytokines such as IL-6, IL-8, TNF-α, and TGF-β (Bhat et al., 2021), and chronic inflammation linked to carcinogen exposure. ECM remodeling and integrin signaling enhance IL-6/TGF-β–mediated STAT3 activation, promoting immunosuppression; Indian GB2-like tumors exhibit stronger stromal–inflammatory programs, consistent with increased resistance compared to less stromal tumors. Conversely, HPV-negative tumors, which are more common in Indian and Asian HNSCCs, tend to have a non-inflamed “cold” tumor microenvironment with limited effective cytotoxic activity despite inflammatory signaling. In addition to immune cell composition, metabolic reprogramming also influences the tumor microenvironment. Metabolically, these tumors exhibit lactate accumulation at the tumor periphery, contributing to immune suppression and altered stromal and immune cell interactions that further inhibit anti-tumor responses. Collectively, these features create a paradoxical TME—inflamed yet ineffective—limiting responses to therapy.

### 4.2 Western immune landscape (predominantly HPV+ve HNSCC)

**FIGURE 1:**
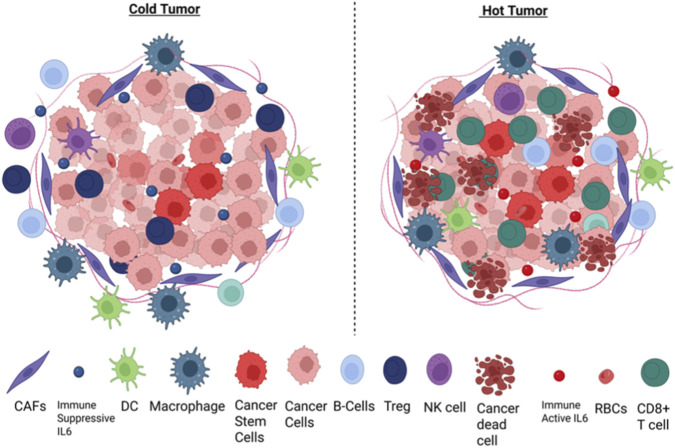
Hot and cold tumor immune microenvironments in HNSCC. Schematic illustration of immune “hot” and “cold” tumor phenotypes in head and neck squamous cell carcinoma (HNSCC). Immune-inflamed (“hot”) tumors are characterized by high infiltration of CD8^+^ cytotoxic T cells, activated NK cells, and antigen-presenting cells. In contrast, immune-desert or immune-excluded (“cold”) tumors show limited T cell infiltration, impaired antigen presentation (e.g., reduced MHC class I expression), and dominance of immunosuppressive components such as M2-polarized macrophages, myeloid-derived suppressor cells (MDSCs), and inhibitory cytokines. These tumors are generally less responsive to immunotherapy and may require combination strategies to induce immune activation and improve therapeutic outcomes (BioRender.com, 2025).

Unlike Indian cohorts, Western groups exhibit a higher frequency of HPV-positive tumors, which significantly affect the tumor microenvironment. In addition to immune cell infiltration, the cytokine environment also influences the TME. HPV-positive HNSCCs typically have an inflamed or “hot” tumor microenvironment characterized by increased infiltration of cytotoxic T cells (Figure 1). These tumors contain more CD3^+^, CD4^+^, and CD8^+^ T cells, CD56dim NK cells, antigen-presenting cells (APCs), myeloid-derived suppressor cells (MDSCs), and dendritic cells (DCs), but fewer Treg cells than HPV-negative tumors (Wang et al., 2023). HPV-positive tumors also exhibit elevated levels of IL-10, CCL2, IL-6, TGF-β, TNF-α, and EGF, which can promote tumor growth and foster an immunosuppressive environment. TGF-β and CCL2 direct macrophage polarization toward the M2 (pro-tumorigenic) phenotype. Nonetheless, strong cytotoxic immune infiltration supports enhanced anti-tumor responses. Western cohorts also show high CD8^+^ T cell infiltration and favorable Treg (regulatory T cell) to CD8^+^ T cell ratios, which are associated with better treatment outcomes (Mandal et al., 2016). These immune cells display signs of activation along with exhaustion. The presence of exhaustion markers such as PD-1, CTLA-4, TIM-3, and LAG-3 on T cells indicates potential responsiveness to checkpoint blockade and underscores the importance of their spatial distribution within the TME (Bhat et al., 2021; Canning et al., 2019). As with immune composition, metabolic adaptations also distinguish HPV-positive tumors. Metabolically, these tumors show increased oxidative phosphorylation in the core and glycolysis in the periphery, reflecting metabolic heterogeneity that supports both tumor growth and immune activity (Krupar et al., 2014). Overall, these features define a highly immunogenic yet regulated tumor microenvironment that underpins improved therapeutic responses in Western HNSCC.

## 5 Approved immunotherapies in Indian and Western populations

**FIGURE 2:**
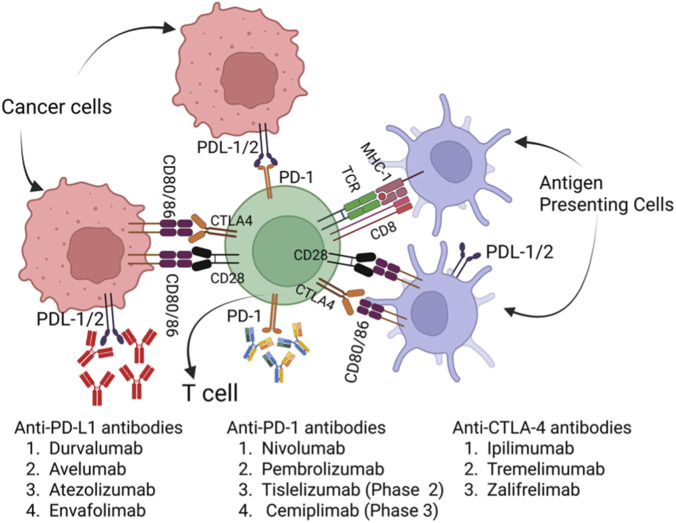
Immune checkpoint blockade mechanisms and combination therapeutic strategies in HNSCC. Immune checkpoint inhibitors boost anti-tumor immunity by blocking inhibitory pathways such as PD-1/PD-L1 and CTLA-4, thereby restoring T cell activation and effector functions within the tumor microenvironment. However, their effectiveness is often limited by tumor-intrinsic and immune-evasive mechanisms, including impaired MHC class I antigen presentation and the expression of nonclassical HLA molecules, such as HLA-G, that engage inhibitory receptors. Importantly, APCs can also present extracellular tumor antigens on MHC-I through cross-presentation, activating CD8^+^ T cells and linking innate and adaptive immunity. In virally associated HNSCC (e.g., HPV-positive tumors), APCs may present viral antigens via both MHC-I and MHC-II pathways. The CTLA-4 pathway is depicted for completeness, but remains investigational in HNSCC.

Immune checkpoint inhibitors targeting PD-1 and PD-L1 have become key treatment options for HNSCC (Figure 2). Drugs like nivolumab and pembrolizumab have shown overall survival (OS) benefits in patients with recurrent or metastatic HNSCC in clinical trials (Checkmate 141 and Keynote clinical trials studies) (Allurkar et al., 2025; Forster and Devlin, 2018). These agents were initially approved in Western populations (FDA/EMA approvals starting in 2016) and were later introduced in India with a delay, as second-line, mainly palliative, agents for recurrent or metastatic settings. In India, both nivolumab and pembrolizumab are approved for clinical use and included in treatment plans for recurrent or metastatic disease (Abbas et al., 2021). However, retrospective data from Indian cohorts indicate slightly lower survival rates than those reported in Western trials. For example, an Indian retrospective study of nivolumab reported a median progression-free survival (PFS) of approximately 2 months and a median overall survival (OS) of about 5 months, with response outcomes including 15% partial responses, 10% stable disease, and a high percentage (75%) of patients experiencing disease progression (Abbas et al., 2021). In contrast, Western clinical trials reported longer median OS values, reaching up to 13.1 months in selected patient populations (Allurkar et al., 2025; Forster and Devlin, 2018). One important consideration is that, in Indian studies, immunotherapy agents were primarily administered as second-line therapy after disease progression, which may contribute to poorer survival outcomes compared to earlier use or more carefully selected patient populations in Western trials. Furthermore, Western studies have often included biomarker assessments, such as PD-L1 expression and p16 status, whereas such evaluations were largely absent in retrospective Indian cohorts, potentially leading to suboptimal patient selection and response heterogeneity (Abbas et al., 2021; Allurkar et al., 2025). These differences may result from variations in treatment timing, biomarker-guided patient selection, and tumor biology. Additionally, agents such as the PD-L1 inhibitor durvalumab, combined with innovative strategies like integrating cytotoxic chemotherapy or radiotherapy (as an *in situ* vaccine), are currently being studied in international trials (Manca et al., 2019).

### 5.1 Ongoing immunotherapy studies in HNSCC

Ongoing immunotherapy studies in HNSCC mainly focus on combining immune checkpoint inhibitors (e.g., anti–PD-1/PD-L1) (Table 2) with radiotherapy, chemotherapy, vaccines, and new immune targets to improve response rates and overcome resistance.

**TABLE 2:** Summary of ongoing clinical trials investigating immunotherapies for head and neck squamous cell carcinoma (HNSCC).

Study title	Interventions	Primary outcome measures	Phases	NCT number
Brachytherapy with radiotherapy and immunotherapy: Guided HDR trial in esophageal squamous cell carcinoma	Nivolumab (240 mg), brachytherapy	Cumulative incidence of locoregional failure at 12 months	PHASE2	NCT07152678
Phase III non-inferiority trial: Reduced-target vs. Full-Target IMRT after chemo in immunotherapy-treated metastatic nasopharyngeal cancer	Reduced-target radiotherapy, conventional full-target radiotherapy	Progression-free survival (PFS)	PHASE3	NCT07188584
E7 T-cell receptor (TCR) -T cell induction therapy for locoregionally advanced HPV-associated cancers	E7 TCR-T cells, aldesleukin	Feasibility of administering E7 TCR-T cell therapy as induction treatment for locally advanced head and neck squamous cell carcinoma, HPV+ve	PHASE2	NCT05639972
Surufatinib in combination with neoadjuvant chemo-immunotherapy and concurrent chemoradiotherapy for patients with unresectable locally advanced esophageal squamous cell carcinoma	Neoadjuvant immunochemotherapy, surufatinib administration, concurrent chemotherapy, radiotherapy	Progression-free survival (PFS) rate	PHASE2	NCT07086469
A clinical trial comparing low-dose RT + targeted Therapy + immunotherapy vs. targeted Therapy + immunotherapy alone as neoadjuvant therapy in operable HNSCC patients	Tislelizumab, afatinib, low dose radiotherapy	Major pathologic response (MPR)	PHASE2	NCT07040956
A clinical trial of neoadjuvant targeted therapy immunotherapy and lysogenic HSV-based virotherapy in resectable head and neck squamous cell carcinoma	Tislelizumab, afatinib, lysogenic HSV virus	Dose-limiting toxicity (DLT)	PHASE1,PHASE2	NCT07010120
Phase 3 study of PDS0101 and pembrolizumab in HPV16+ recurrent/Metastatic head and neck squamous cell carcinoma	PDS0101 and pembrolizumab, pembrolizumab monotherapy	Overall survival (OS)	PHASE3	NCT06790966
Towards cure via only ultra-short ICB in CSCC	Nivolumab, ipilumimab	Rate of clinical complete remission after only immunotherapy	PHASE2	NCT06823479
QL1706 (PD-1/CTLA-4 Bi-specific antibody) and chemoradiotherapy in locoregionally-advanced nasopharyngeal carcinoma	QL1706, gemcitabine, cisplatin, intensity-modulated radiotherapy	Failure-free survival (FFS)	PHASE3	NCT06749899
Intestinal low dose radiotherapy combined with immunotherapy in immune-resistant metastatic malignant solid tumors	Low-dose radiotherapy to the intestine (ILDR), PD-1/PD-l1 monoclonal antibodies	Objective response rate (ORR) disease control rate (DCR), progression free survival (PFS)	PHASE2	NCT07071103
Hypofractionated vs. Conventional chemoradiotherapy after induction chemo-immunotherapy for unresectable esophageal squamous cell carcinoma	Induction chemoimmunotherapy, hypofractionated radiation therapy, conventional fractionated radiation therapy, concurrent chemotherapy	Progression-free survival (PFS) rate	PHASE2	NCT06912074
A phase II study of toripalimab combined with sequential neoadjuvant chemoradiotherapy in patients with esophageal squamous cell carcinoma	Toripalimab (240 mg day1 Q3W*3cycle)	Pathological complete response rate (pCR)	PHASE2	NCT06843889
Two-cycle and three-cycle induction therapy with modified TPF regimen combined and camrelizumab for LANPC	Two-cycle induction chemotherapy + immunotherapy, three-cycle induction chemotherapy + immunotherapy	Complete response	PHASE2	NCT06811844
Cetuximab plus platinum and taxane-based chemotherapy followed by avelumab and cetuximab as first-line treatment for recurrent/Metastatic head and neck squamous cell carcinoma (HNSCC) patients with a PD-L1 combined positive score (CPS) ≥ 1 ≤ 19	Cetuximab/avelumab	6-months (6m)-progression free survival (PFS)	PHASE2	NCT06869473
Lymphocyte-sparing and radio-immunotherapy in head and neck carcinoma	Vesanoid, standard radiotherapy, tailored radiotherapy, cisplatin, cetuximab	Event free survival (EFS)	PHASE3	NCT06706401
Testing the addition of an immunotherapy drug cemiplimab (REGN2810) plus surgery to the usual surgery alone for treating advanced skin cancer	Cemiplimab, image guided radiation therapy, intensity-modulated radiation therapy, magnetic resonance imaging, positron emission tomography	Event-free survival (EFS)	PHASE3	NCT06568172
FORTIFI-HN01: A study of ficerafusp alfa (BCA101) or placebo in combination with pembrolizumab in first-line PD-l1-pos R or M HNSCC	Ficerafusp alfa, pembrolizumab (KEYTRUDA®)	Objective response rate (ORR), overall survival (OS)	PHASE2,PHASE3	NCT06788990
Neoadjuvant low-dose radiotherapy plus targeted-immunotherapy vs. targeted-immunotherapy monotherapy in resectable HNSCC: A randomized trial	Tislelizumab, afatinib, low dose radiotherapy	Major pathologic response (MPR)	PHASE2	NCT06804850
LDRT and chemoimmunotherapy in NPC with liver metastasis	Low-dose radiotherapy combine with chemoimmunotherapy	Intrahepatic progression-free survival (iPFS)	PHASE2	NCT06788002
A study of evofosfamide in combination with zalifrelimab and balstilimab	Evofosfamide, zalifrelimab, balstilimab	Dose-limiting toxicity (DLT)	PHASE1,PHASE2	NCT06782555

## 6 Population-specific differences in MHC alleles and immunotherapeutic response in Indian and Western HNSCC patients

**FIGURE 3:**
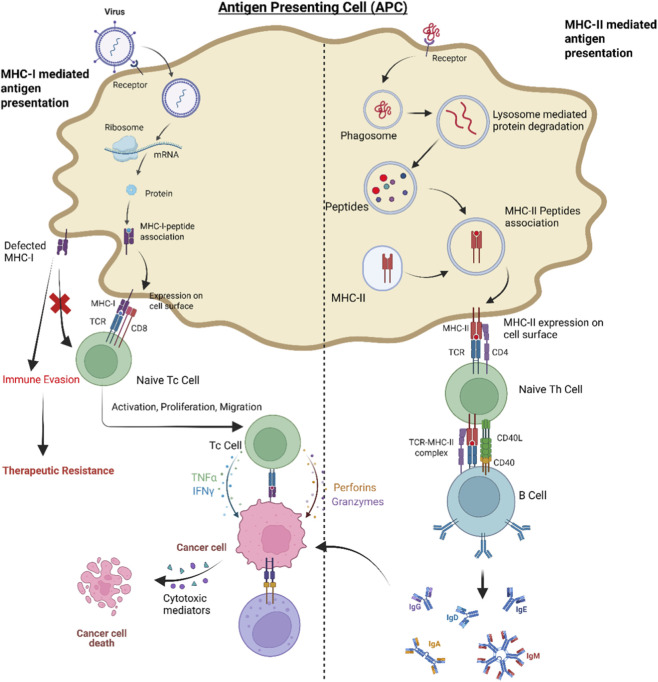
Role of MHC class I and II antigen presentation pathways in shaping anti-tumor immunity in HNSCC. A schematic showing how antigen presentation through MHC class I and II pathways influences anti-tumor immune responses in head and neck squamous cell carcinoma (HNSCC). MHC class I molecules display intracellular tumor-derived peptides to CD8^+^ cytotoxic T cells, while MHC class II molecules present extracellular antigens to CD4^+^ helper T cells, collectively coordinating adaptive immunity. Genetic variation in HLA alleles among populations affects peptide binding, antigen presentation efficiency, and subsequent T cell activation. These population-specific HLA polymorphisms contribute to differences in immune responses, recognition, and modulation of responsiveness to immunotherapies, highlighting their crucial role in shaping patient-specific anti-tumor immunity.

Emerging evidence shows that population-specific variation in major histocompatibility complex (MHC) alleles significantly affects immunotherapeutic responses in head and neck squamous cell carcinoma (HNSCC). MHC molecules are essential for antigen presentation and activating adaptive immune responses (Figure 3), with their high diversity across populations leading to differences in tumor antigen recognition and immune responsiveness (Goel et al., 2022; Manca et al., 2019). While professional antigen-presenting cells are vital for MHC-mediated immunity, tumor cells also actively present antigens through MHC-I. In HNSCC, treatments like radiotherapy and immune checkpoint blockade can increase tumor cell–intrinsic MHC-I expression and antigen processing, thus enhancing CD8^+^ T-cell recognition and cytotoxic responses. However, defects in the MHC-I pathway in cancer cells remain a key mechanism of immune evasion within the tumor microenvironment. Variations in MHC allelic types between Indian and Western populations may influence tumor-infiltrating lymphocyte (TIL) density, antigen presentation efficiency, and overall immune response to therapy (von Witzleben et al., 2020).

### 6.1 Indian population

Research on MHC-associated immunotherapy response in Indian populations remains limited but is gaining momentum. Epigenetic studies in Indian oral squamous cell carcinoma (OSCC) suggest that environmental exposures—such as tobacco use and betel quid chewing—interact with genetic predispositions to influence MHC expression and antigen presentation profiles (Basu et al., 2017). Population-specific HLA allele distributions have been reported in Indian HNSCC cohorts. A recent study demonstrated that HLA-A02, HLA-A24, and HLA-A33 is more frequently observed in patients, whereas HLA-A01 is significantly reduced and HLA-A24 is significantly increased (Shahab and Ahluwalia, 2023). Furthermore, HLA-A02 is associated with advanced-stage tumors (III/IV), while HLA-A03 is more common in early-stage disease (I/II), suggesting a role in disease progression and tumor immune dynamics (Shahab and Ahluwalia, 2023). Functional studies also support the importance of allele-specific immune responses. For example, an EGFR-derived epitope (EGFR875–889) elicited strong CD4^+^ T cell responses mediated by HLA-DR alleles prevalent in Indian populations (Kumai et al., 2013). Collectively, these findings indicate that Indian-specific MHC variability may influence tumor microenvironment composition, T cell activation, and immunotherapy outcomes. However, large-scale, comprehensive studies in Indian and South Asian populations remain limited, revealing a significant gap in current research.

### 6.2 Western populations

In contrast, Western populations have been extensively studied, with strong evidence linking specific HLA alleles to HNSCC susceptibility and immune responses. Genome-wide.

Genome-wide association studies (GWAS) have identified significant links between HNSCC risk and HLA class II alleles, especially HLA-DRB1*1301, HLA-DQA1*0103, and HLA-DQB1*0603 (Shete et al., 2020). Higher HLA-DQB1 mRNA levels are associated with a reduced risk of oropharyngeal cancer, underscoring its role in tumor immune defense (Kumai et al., 2013; Shete et al., 2020). Moreover, polymorphisms at various HLA loci—including HLA-B, HCP5, HLA-DRA, HLA-DRB1, HLA-DQA1, and HLA-DQB1—have been associated with susceptibility to HPV-related and other cancers (Lesseur et al., 2016; Zhang et al., 2015; Wu et al., 2002). Mechanistic studies also reveal immune evasion tactics involving MHC dysfunction. Defects in MHC class I antigen processing hinder recognition by CD8^+^ T cells, enabling immune escape and therapy resistance (Charap et al., 2020; Gao et al., 2022). Additionally, nonclassical MHC molecules such as HLA-G, through interactions with inhibitory receptors ILT2 and ILT4, foster an immunosuppressive tumor microenvironment and reduce responses to checkpoint inhibitors (Durmanova et al., 2024; Zervanos et al., 2025).

### 6.3 Comparative perspective

While both Indian and Western populations exhibit significant MHC polymorphism affecting HNSCC biology, the extent of research differs markedly. Western populations have access to extensive genomic and functional datasets, whereas Indian research remains somewhat limited but uncovers unique allele patterns and gene–environment interactions (Mishra and Meherotra, 2019). These differences could result in varying tumor immune microenvironments and responses to immunotherapy. Therefore, increasing research in populations, especially in underrepresented groups such as Indian cohorts, is essential for developing fair and effective precision immunotherapy strategies.

## 7 Epigenetic regulators influencing MHC-I expression and antigen presentation

The current standard of care for HNSCC offers subpar survival rates and is associated with long-term toxicities. Although immune checkpoint inhibitors targeting the PD-1/PD-L1 axis have improved outcomes for some patients, many HNSCC tumors remain resistant to immunotherapy. One mechanism of immune escape involves the epigenetic suppression of antigen presentation pathways. Several studies demonstrate that epigenetic repression can hinder MHC class I (MHC-I) antigen presentation, thereby facilitating immune evasion and cancer cell growth. For example, the histone methyltransferase EZH2, a key component of the Polycomb Repressive Complex 2 (PRC2), is negatively associated with antigen processing machinery. Inhibiting EZH2 or knocking out its gene increases MHC-I expression, which enhances antigen-specific CD8^+^ T-cell proliferation and IFN-γ production by reducing the repressive histone mark H3K27me3 at B2M promoters. This also makes tumors resistant to anti-PD-1 therapy more responsive to immune checkpoint blockade (Zhou et al., 2020; Burkitt and Saloura, 2021). Meanwhile, histone acetyltransferases and deacetylases regulate chromatin structure and gene transcription; acetylation relaxes chromatin, making genes more accessible for transcription, whereas deacetylation tightens chromatin. Both histone deacetylase (HDAC) and DNA methyltransferase inhibitors have been shown to increase MHC/HLA expression and enhance antigen processing machinery. HDACs control several essential cellular processes, including cell cycle progression, apoptosis, DNA damage response, metastasis, and angiogenesis, underscoring the therapeutic potential of targeting epigenetic regulators to strengthen antitumor immunity in HNSCC (Li et al., 2020).

A comprehensive genomic analysis from the International Cancer Genome Consortium India oral squamous cell carcinoma cohort (OSCC-GB) provides important insights into population-specific tumor biology. This study confirmed common genetic alterations shared with global HNSCC (e.g., TP53, FAT1, NOTCH1, HRAS, CASP8) while emphasizing genes more frequently altered in Indian OSCC, including USP9X, MLL4 (KMT2D), and ARID2—many of which are key regulators of chromatin remodeling and epigenetic mechanisms. Notably, tumors from individuals who chew tobacco showed a higher load of C>G transversions, linking environmental factors to distinct mutational signatures. The analysis also identified molecular subgroups (e.g., CASP8±FAT1 patterns), revealing biologically distinct disease subsets. These findings demonstrate a convergence of genetic and epigenetic dysregulation driven by carcinogen exposure, with implications for tumor development, immune response, and therapy in Indian HNSCC (India Project Team of ICGC, 2013; Shen et al., 2015).

## 8 Radiotherapy in Indian and Western HNSCC populations

Indian patients with oropharyngeal squamous cell carcinoma treated with radiotherapy show only 11% HPV positivity. HPV-positive patients have significantly better outcomes than HPV-negative patients, including 5-year local control (84.4% vs. 43.5%, p-value <0.001), loco-regional control (71.3% vs. 31.8%, p-value < 0.001), disease-free survival (63.9% vs. 26.1%, p-value <0.0001), and overall survival (69.1% vs. 31.9%, p-value <0.001) (Budrukkar et al., 2025). Large Western clinical trials and cohort studies consistently show that HPV-positive HNSCC is significantly more radiosensitive, with overall survival (OS) around 70%–85% in HPV + patients compared to approximately 30%–50% in HPV− patients. Additionally, there is a notable improvement in progression-free survival (PFS), with about 80%–90% of HPV + patients demonstrating locoregional control and a better response to chemoradiotherapy. Although the prevalence of HPV-positive HNSCC is much higher in Western populations than in Indian and Asian populations (Murthy et al., 2017), HPV status remains a critical prognostic biomarker regardless of geographic location. Radiotherapy not only causes direct tumor cell death but also modulates the tumor microenvironment by enhancing antigen presentation, promoting immune cell infiltration, activating innate immune signaling pathways, and inducing immunosuppressive mediators such as TGF-β (Barker et al., 2015; Deng et al., 2014). It also acts as an *in situ* vaccine by triggering immunogenic cell death and activating tumor-specific T-cell responses, thereby working synergistically with checkpoint inhibitors such as pembrolizumab and nivolumab to counteract TME-mediated immunosuppression and enhance therapeutic efficacy (Sharabi et al., 2015). As a result, the higher prevalence of immunogenic, HPV-positive tumors in Western populations supports both better radiotherapy responses and the move toward treatment reduction using combined RT–immunotherapy strategies.

## 9 Challenges

Despite significant progress in understanding the tumor microenvironment (TME) and immune landscape in HNSCC, several challenges remain. A key obstacle is the limited availability of high-quality, comprehensive data, especially from Indian cohorts. Most current research relies on TCGA datasets, which primarily represent Western populations, thereby limiting insights into the unique molecular and immunological features of Indian and broader Asian populations. Additionally, the considerable tumor heterogeneity seen in both Western and Indian tumors complicates efforts to develop universal treatment strategies. Variations in the expression of immunoregulatory markers, differences in immune cell composition, and distinct metabolic adaptations within tumors all contribute to complex interactions that influence treatment outcomes. Addressing this complexity will require continued use of advanced technologies such as single-cell RNA sequencing, spatial transcriptomics, and organoid cultures, which provide detailed insights into the TME and can help identify new therapeutic targets (Welters et al., 2018).

## 10 Conclusion

A comparative analysis of the tumor microenvironment and immune landscape between Western and Indian (or Asian) head and neck cancer patients highlights both shared and population-specific differences. Traits influenced by environmental factors, genetic predisposition, and viral infections are evident. The differences observed in immune cell infiltration, cytokine profiles, metabolic pathways, and immunoregulatory molecule expression suggest that a single, universal treatment approach may not be sufficient. The ongoing challenge is to use these insights to develop strong, population-specific therapeutic strategies that improve patient outcomes worldwide. As new immunotherapeutic agents emerge and multi-omics technologies become more accessible, the potential for personalized, precision treatments for head and neck cancer grows more promising. MHC alleles play a crucial role in the immune response to HNSCC. Future efforts should focus on integrating multi-omics data to gain a comprehensive understanding of the MHC landscape, address existing knowledge gaps, and ultimately develop tailored immunotherapy approaches.
